# Supplementary figures and images for: Cryopreservation of Human Mucosal Leukocytes
Source: PLoS One. 2016 May 27;11(5):e0156293. doi: 10.1371/journal.pone.0156293 (PMC4883784; doi:10.1371/journal.pone.0156293)

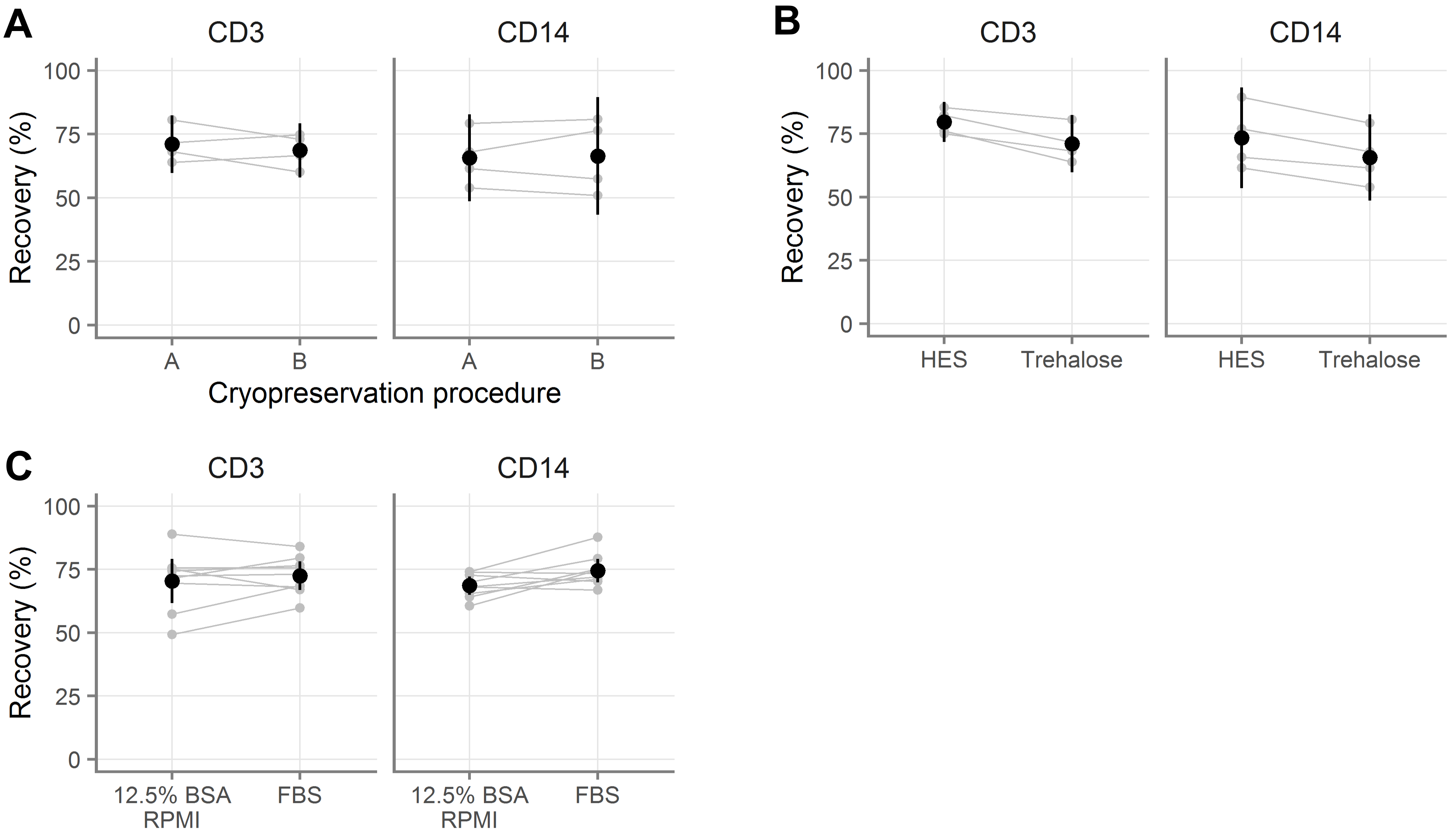

Supplement: S1 Fig — A, Comparison of different procedures (see Table 1) for cryopreserving vaginal T cells (indicated “CD3”) and macrophages (indicated “CD14”). B, Comparison of supplementing 5% EG and 6% DMSO in FBS with 6% HES or 1.2% trehalose. C, Comparison of cryopreservation with 5% EG, 6% DMSO, and 6% HES in FBS or in 12.5% BSA in RPMI. Gray symbols indicate the average of duplicates, with gray lines indicating pairing. Black symbols show the mean across all samples and black vertical lines show the 95% confidence interval of the mean. (TIFF) [file pone.0156293.s001.tiff]
