# Supplementary material for: Cryopreservation of Human Mucosal Leukocytes
Source: PLoS One. 2016 May 27;11(5):e0156293. doi: 10.1371/journal.pone.0156293 (PMC4883784; doi:10.1371/journal.pone.0156293)
Supplement: S1 File — (PDF) [file pone.0156293.s002.pdf]

Cryopreservation of human mucosal immune cells: statistics

Contents

Validation of novel cryopreservation medium **2**

Repeated measures ANOVAs with Tukey post-test (parametric, paired) 3

Correlations 3

Comparison with published cryopreservation methods **4**

Comparison with published methods: Viability and recovery 4

Comparison with published methods: Repeated measures ANOVA with Tukey post-test 5

Optimal processing **7**

Which steps matter? 7

HANC rescue: viability, recovery, and effect sizes 9

Cryopreservation of endocervical cytobrushes **13**

Cryopreservation of whole cytobrushes **13**

Cryopreservation of colorectal immune cells **15**

Validation of novel cryopreservation medium

Table A: Relative viability

CellType	Condition	Mean	95% CI	n
CD3	DT	78.87	[73.88, 83.87]	10
CD3	Mix	84.20	[79.41, 89]	10
CD3	10D	72.82	[66.53, 79.11]	10
CD14	DT	82.41	[77.98, 86.85]	10
CD14	Mix	77.23	[69.81, 84.66]	10
CD14	10D	79.10	[71.48, 86.71]	10

Table B: Recovery

CellType	Condition	Mean	95% CI	n
CD3	DT	60.03	[49.51, 70.56]	10
CD3	Mix	68.03	[56.43, 79.64]	10
CD3	10D	52.56	[43.35, 61.76]	10
CD14	DT	72.89	[62.97, 82.82]	10
CD14	Mix	67.54	[57.81, 77.27]	10
CD14	10D	67.94	[59.38, 76.49]	10

Table C: Viability effect size (percent difference from 10% DMSO)

CellType	Condition	Mean	95% CI	n
CD3	DT	6.053	[1.95, 10.16]	10
CD3	Mix	11.383	[7.87, 14.9]	10
CD14	DT	3.317	[-0.33, 6.96]	10
CD14	Mix	-1.862	[-3.35, -0.37]	10

Table D: Recovery effect size (percent difference from 10% DMSO)

CellType	Condition	Mean	95% CI	n
CD3	DT	7.4752	[2.04, 12.91]	10
CD3	Mix	15.4770	[8.78, 22.17]	10
CD14	DT	4.9578	[-0.18, 10.09]	10
CD14	Mix	-0.3973	[-5.42, 4.62]	10

Repeated measures ANOVAs with Tukey post-test (parametric, paired)

Table E: CD3 viability, repeated measures ANOVA with Tukey post-test

Comparisons	Estimate	Std. Error	Z value	p
10D - DT	-6.05	1.5	-4.04	1.56e-04
10D - Mix	-11.38	1.5	-7.6	4.55e-14
Mix - DT	5.33	1.5	3.56	1.06e-03

Table F: CD14 viability, repeated measures ANOVA with Tukey post-test

Comparisons	Estimate	Std. Error	Z value	p
10D - DT	-3.32	1.33	-2.5	0.03
10D - Mix	1.86	1.33	1.4	0.34
Mix - DT	-5.18	1.33	-3.9	2.66e-04

Table G: CD3 recovery, repeated measures ANOVA with Tukey post-test

Comparisons	Estimate	Std. Error	Z value	p
10D - DT	-7.48	2.45	-3.05	6.59e-03
10D - Mix	-15.48	2.45	-6.31	2.91e-09
Mix - DT	8	2.45	3.26	3.22e-03

Table H: CD14 recovery, repeated measures ANOVA with Tukey post-test

Comparisons	Estimate	Std. Error	Z value	p
10D - DT	-4.96	2.21	-2.24	0.06
10D - Mix	0.4	2.21	0.18	0.98
Mix - DT	-5.36	2.21	-2.42	0.04

Correlations

Table I: Recovery as a function of viability

CellType	Condition	AdjustedR^2	Coef	p
CD3	DT	0.388086	1.4240	0.03
CD3	Mix	0.336378	1.5490	0.05
CD3	10D	0.369375	0.9704	0.04
CD14	DT	0.001662	-0.7512	0.34
CD14	Mix	-0.101937	-0.1876	0.69
CD14	10D	-0.074027	-0.2389	0.55

Comparison with published cryopreservation methods

Table J: Comparison of cryopreservation procedures. Effect size relative to procedure B, paired t-test

CellType	Condition	Mean	95% CI	p	n
CD3	Proc A	-2.4397	[-12.32, 7.44]	0.49	4
CD14	Proc A	0.7515	[-8.28, 9.79]	0.81	4

Table K: Comparison of 1.2% trehalose and 6% HES. Effect size relative to trehalose, paired t-test

CellType	Condition	Mean	95% CI	p	n
CD3	HES	8.657	[3.24, 14.07]	0.01	4
CD14	HES	7.760	[3.61, 11.91]	9.47e-03	4

Comparison with published methods: Viability and recovery

Table L: Absolute viability

CellType	Condition	Mean	95% CI	n
CD3	GHRC I	78.88	[72.18, 85.57]	6
CD3	GHRC II	77.56	[70.55, 84.57]	6
CD3	HANC	75.52	[68.53, 82.5]	6
CD3	Seattle	78.72	[70.56, 86.87]	6
CD14	GHRC I	82.41	[73.56, 91.25]	6
CD14	GHRC II	81.81	[72.36, 91.25]	6
CD14	HANC	78.41	[67.72, 89.1]	6
CD14	Seattle	84.58	[75.27, 93.89]	6

Table M: Recovery

CellType	Condition	Mean	95% CI	n
CD3	GHRC I	77.48	[69.96, 85]	6
CD3	GHRC II	80.40	[73.64, 87.15]	6
CD3	HANC	67.42	[60.9, 73.94]	6
CD3	Seattle	80.31	[75.74, 84.88]	6
CD14	GHRC I	73.32	[63.35, 83.29]	6
CD14	GHRC II	77.21	[69.43, 85]	6
CD14	HANC	59.74	[51.01, 68.48]	6
CD14	Seattle	75.99	[67.5, 84.48]	6

Comparison with published methods: Repeated measures ANOVA with Tukey post-test

Table N: CD3 viability, repeated measures ANOVA with Tukey post-test

Comparisons	Estimate	Std. Error	Z value	p
GHRC II - GHRC I	-1.32	0.77	-1.72	0.32
HANC - GHRC I	-3.36	0.77	-4.38	5.02e-05
HANC - GHRC II	-2.04	0.77	-2.66	0.04
Seattle - GHRC I	-0.16	0.77	-0.21	1
Seattle - GHRC II	1.16	0.77	1.51	0.43
Seattle - HANC	3.2	0.77	4.17	1.58e-04

Table O: CD14 viability, repeated measures ANOVA with Tukey post-test

Comparisons	Estimate	Std. Error	Z value	p
GHRC II - GHRC I	-0.6	2.15	-0.28	0.99
HANC - GHRC I	-4	2.15	-1.86	0.25
HANC - GHRC II	-3.4	2.15	-1.58	0.39
Seattle - GHRC I	2.18	2.15	1.01	0.74
Seattle - GHRC II	2.77	2.15	1.29	0.57
Seattle - HANC	6.18	2.15	2.87	0.02

Table P: CD3 recovery, repeated measures ANOVA with Tukey post-test

Comparisons	Estimate	Std. Error	Z value	p
GHRC II - GHRC I	2.92	2.41	1.21	0.62
HANC - GHRC I	-10.06	2.41	-4.18	1.96e-04
HANC - GHRC II	-12.98	2.41	-5.4	3.04e-07
Seattle - GHRC I	2.83	2.41	1.18	0.64
Seattle - GHRC II	-0.09	2.41	-0.04	1
Seattle - HANC	12.89	2.41	5.36	3.01e-07

Table Q: CD14 recovery, repeated measures ANOVA with Tukey post-test

Comparisons	Estimate	Std. Error	Z value	p
GHRC II - GHRC I	3.9	2.48	1.57	0.39
HANC - GHRC I	-13.57	2.48	-5.48	1.28e-07
HANC - GHRC II	-17.47	2.48	-7.05	5.83e-12
Seattle - GHRC I	2.67	2.48	1.08	0.7
Seattle - GHRC II	-1.22	2.48	-0.49	0.96
Seattle - HANC	16.25	2.48	6.56	1.23e-10

Table R: Comparison of cryopreservation in FBS or 12.5% BSA in RPMI, both with 6% DMSO, 5% EG, and 6% HES. Effect size relative to FBS, paired t-test

CellType	Condition	Mean	95% CI	p	n
CD3	BSA-RPMI	-2.039	[-7.21, 3.13]	0.39	9
CD14	BSA-RPMI	-5.935	[-10.89, -0.98]	0.02	9

Optimal processing

Which steps matter?

Table S: Absolute viability

CellType	Condition	Mean	95% CI	n
CD3	HANC	71.42	[67.19, 75.65]	5
CD3	Slow addition	70.19	[66.56, 73.82]	5
CD3	No benzonase	70.74	[66.27, 75.21]	5
CD3	15 mL tube	70.70	[67.21, 74.19]	5
CD3	1 wash	68.25	[62.5, 74]	5
CD14	HANC	86.26	[78.33, 94.19]	5
CD14	Slow addition	85.99	[77.28, 94.7]	5
CD14	No benzonase	85.76	[77.74, 93.78]	5
CD14	15 mL tube	83.94	[75.15, 92.73]	5
CD14	1 wash	82.60	[74.35, 90.85]	5

Table T: Average recovery

CellType	Condition	Mean	95% CI	n
CD3	HANC	60.14	[43.06, 77.22]	5
CD3	Slow addition	61.05	[47.04, 75.06]	5
CD3	No benzonase	62.19	[48.95, 75.42]	5
CD3	15 mL tube	62.45	[46.69, 78.2]	5
CD3	1 wash	68.22	[55.98, 80.45]	5
CD14	HANC	50.08	[30.47, 69.69]	5
CD14	Slow addition	50.98	[29.35, 72.6]	5
CD14	No benzonase	51.87	[33.93, 69.81]	5
CD14	15 mL tube	56.84	[36.69, 76.99]	5
CD14	1 wash	58.14	[45.81, 70.48]	5

Table U: Viability relative to HANC

CellType	Condition	Mean	95% CI	n
CD3	Slow addition	-1.6268	[-3.1, -0.15]	5
CD3	No benzonase	-0.8892	[-3.59, 1.81]	5
CD3	15 mL tube	-0.8521	[-6.35, 4.64]	5
CD3	1 wash	-4.2081	[-7.58, -0.84]	5
CD14	Slow addition	-0.2848	[-2.67, 2.1]	5
CD14	No benzonase	-0.5654	[-3.5, 2.37]	5
CD14	15 mL tube	-2.4807	[-4.82, -0.14]	5
CD14	1 wash	-3.8997	[-9.56, 1.76]	5

Table V: Recovery relative to HANC

CellType	Condition	Mean	95% CI	n
CD3	Slow addition	0.9059	[-3.77, 5.58]	5
CD3	No benzonase	2.0426	[-2.6, 6.69]	5
CD3	15 mL tube	2.3026	[-3.59, 8.19]	5
CD3	1 wash	8.0725	[0.53, 15.62]	5
CD14	Slow addition	0.8960	[-5.68, 7.47]	5
CD14	No benzonase	1.7912	[-3.02, 6.6]	5
CD14	15 mL tube	6.7582	[-0.8, 14.31]	5
CD14	1 wash	8.0639	[-0.67, 16.79]	5

Table W: CD3 viability, repeated measures ANOVA with Tukey post-test

Comparisons	Estimate	Std. Error	Z value	p
1 wash - 15 mL tube	-2.45	1.22	-2	0.26
1 wash - HANC	-3.17	1.22	-2.59	0.07
1 wash - No benzonase	-2.49	1.22	-2.04	0.25
1 wash - Slow addition	-1.94	1.22	-1.59	0.51
15 mL tube - HANC	-0.72	1.22	-0.59	0.98
15 mL tube - No benzonase	-0.04	1.22	-0.03	1
15 mL tube - Slow addition	0.51	1.22	0.42	0.99
No benzonase - HANC	-0.68	1.22	-0.56	0.98
No benzonase - Slow addition	0.55	1.22	0.45	0.99
Slow addition - HANC	-1.23	1.22	-1.01	0.85

Table X: CD14 viability, repeated measures ANOVA with Tukey post-test

Comparisons	Estimate	Std. Error	Z value	p
1 wash - 15 mL tube	-1.34	1.58	-0.85	0.91
1 wash - HANC	-3.66	1.58	-2.32	0.14
1 wash - No benzonase	-3.16	1.58	-2.01	0.26
1 wash - Slow addition	-3.39	1.58	-2.15	0.2
15 mL tube - HANC	-2.32	1.58	-1.47	0.58
15 mL tube - No benzonase	-1.82	1.58	-1.16	0.78
15 mL tube - Slow addition	-2.05	1.58	-1.3	0.69
No benzonase - HANC	-0.5	1.58	-0.32	1
No benzonase - Slow addition	-0.23	1.58	-0.15	1
Slow addition - HANC	-0.27	1.58	-0.17	1

Table Y: CD3 recovery, repeated measures ANOVA with Tukey post-test

Comparisons	Estimate	Std. Error	Z value	p
1 wash - 15 mL tube	5.77	2.15	2.69	0.06
1 wash - HANC	8.07	2.15	3.76	1.56e-03
1 wash - No benzonase	6.03	2.15	2.81	0.04
1 wash - Slow addition	7.17	2.15	3.34	7.34e-03
15 mL tube - HANC	2.3	2.15	1.07	0.82
15 mL tube - No benzonase	0.26	2.15	0.12	1
15 mL tube - Slow addition	1.4	2.15	0.65	0.97
No benzonase - HANC	2.04	2.15	0.95	0.88
No benzonase - Slow addition	1.14	2.15	0.53	0.98
Slow addition - HANC	0.91	2.15	0.42	0.99

Table Z: CD14 recovery, repeated measures ANOVA with Tukey post-test

Comparisons	Estimate	Std. Error	Z value	p
1 wash - 15 mL tube	1.31	2.99	0.44	0.99
1 wash - HANC	8.06	2.99	2.69	0.05
1 wash - No benzonase	6.27	2.99	2.1	0.22
1 wash - Slow addition	7.17	2.99	2.4	0.12
15 mL tube - HANC	6.76	2.99	2.26	0.16
15 mL tube - No benzonase	4.97	2.99	1.66	0.46
15 mL tube - Slow addition	5.86	2.99	1.96	0.29
No benzonase - HANC	1.79	2.99	0.6	0.98
No benzonase - Slow addition	0.9	2.99	0.3	1
Slow addition - HANC	0.9	2.99	0.3	1

HANC rescue: viability, recovery, and effect sizes

Table AA: Absolute viability

CellType	Condition	Mean	95% CI	n
CD3	Frau I	72.43	[67.58, 77.29]	6
CD3	Frau II	71.64	[67.53, 75.75]	6
CD3	HANC	67.57	[62.51, 72.62]	6
CD3	Opt HANC	70.67	[67.13, 74.22]	6
CD3	Seattle	72.84	[69.83, 75.85]	6
CD14	Frau I	84.81	[79.68, 89.94]	6
CD14	Frau II	84.01	[78.68, 89.34]	6
CD14	HANC	79.88	[75.67, 84.08]	6
CD14	Opt HANC	81.49	[77.19, 85.79]	6
CD14	Seattle	84.88	[79.67, 90.1]	6

Table AB: Average recovery

CellType	Condition	Mean	95% CI	n
CD3	Frau I	69.13	[58.28, 79.99]	6
CD3	Frau II	65.67	[53.44, 77.89]	6
CD3	HANC	50.66	[40.17, 61.16]	6
CD3	Opt HANC	64.01	[54.07, 73.95]	6
CD3	Seattle	68.85	[62.87, 74.83]	6
CD14	Frau I	72.40	[62.84, 81.97]	6
CD14	Frau II	69.88	[60.92, 78.84]	6
CD14	HANC	46.25	[37.63, 54.88]	6
CD14	Opt HANC	68.85	[59.67, 78.03]	6
CD14	Seattle	74.42	[66.91, 81.94]	6

Table AC: Viability relative to HANC

CellType	Condition	Mean	95% CI	n
CD3	Frau I	7.644	[2.86, 12.43]	6
CD3	Frau II	5.337	[2.14, 8.53]	6
CD3	HANC	0.000	[0, 0]	6
CD3	Opt HANC	3.933	[1.48, 6.38]	6
CD3	Seattle	6.635	[2.23, 11.04]	6
CD14	Frau I	8.456	[6.35, 10.56]	6
CD14	Frau II	8.500	[4.96, 12.04]	6
CD14	HANC	0.000	[0, 0]	6
CD14	Opt HANC	1.763	[-0.43, 3.96]	6
CD14	Seattle	5.435	[3.47, 7.4]	6

Table AD: Recovery relative to HANC

CellType	Condition	Mean	95% CI	n
CD3	Frau I	18.47	[6.37, 30.57]	6
CD3	Frau II	15.00	[5.16, 24.84]	6
CD3	HANC	0.00	[0, 0]	6
CD3	Opt HANC	13.34	[6.49, 20.2]	6
CD3	Seattle	18.18	[8.84, 27.53]	6
CD14	Frau I	26.15	[10.94, 41.36]	6
CD14	Frau II	23.63	[13.14, 34.11]	6
CD14	HANC	0.00	[0, 0]	6
CD14	Opt HANC	22.60	[12.74, 32.46]	6
CD14	Seattle	28.17	[15.88, 40.46]	6

Table AE: CD3 viability, repeated measures ANOVA with Tukey post-test

Comparisons	Estimate	Std. Error	Z value	p
Frau II - Frau I	-0.79	1.01	-0.79	0.93
HANC - Frau I	-4.87	1.01	-4.84	1.13e-05
HANC - Frau II	-4.07	1.01	-4.05	4.47e-04
Opt HANC - Frau I	-1.76	1.01	-1.75	0.4
Opt HANC - Frau II	-0.97	1.01	-0.96	0.87
Opt HANC - HANC	3.11	1.01	3.09	0.02
Seattle - Frau I	0.41	1.01	0.41	0.99
Seattle - Frau II	1.2	1.01	1.19	0.76
Seattle - HANC	5.27	1.01	5.25	2.86e-06
Seattle - Opt HANC	2.17	1.01	2.16	0.2

Table AF: CD14 viability, repeated measures ANOVA with Tukey post-test

Comparisons	Estimate	Std. Error	Z value	p
Frau II - Frau I	-0.8	0.92	-0.87	0.91
HANC - Frau I	-4.93	0.92	-5.37	5.34e-07
HANC - Frau II	-4.13	0.92	-4.5	5.42e-05
Opt HANC - Frau I	-3.32	0.92	-3.61	2.74e-03
Opt HANC - Frau II	-2.52	0.92	-2.74	0.05
Opt HANC - HANC	1.62	0.92	1.76	0.4
Seattle - Frau I	0.07	0.92	0.08	1
Seattle - Frau II	0.87	0.92	0.95	0.88
Seattle - HANC	5.01	0.92	5.45	5.40e-07
Seattle - Opt HANC	3.39	0.92	3.69	2.05e-03

Table AG: CD3 recovery, repeated measures ANOVA with Tukey post-test

Comparisons	Estimate	Std. Error	Z value	p
Frau II - Frau I	-3.47	3.32	-1.05	0.83
HANC - Frau I	-18.47	3.32	-5.57	1.50e-07
HANC - Frau II	-15	3.32	-4.52	7.13e-05
Opt HANC - Frau I	-5.13	3.32	-1.55	0.53
Opt HANC - Frau II	-1.66	3.32	-0.5	0.99
Opt HANC - HANC	13.34	3.32	4.02	5.59e-04
Seattle - Frau I	-0.29	3.32	-0.09	1
Seattle - Frau II	3.18	3.32	0.96	0.87
Seattle - HANC	18.18	3.32	5.48	3.78e-07
Seattle - Opt HANC	4.84	3.32	1.46	0.59

Table AH: CD14 recovery, repeated measures ANOVA with Tukey post-test

Comparisons	Estimate	Std. Error	Z value	p
Frau II - Frau I	-2.52	3.82	-0.66	0.96
HANC - Frau I	-26.15	3.82	-6.85	3.44e-11
HANC - Frau II	-23.63	3.82	-6.19	3.05e-09
Opt HANC - Frau I	-3.55	3.82	-0.93	0.89
Opt HANC - Frau II	-1.03	3.82	-0.27	1
Opt HANC - HANC	22.6	3.82	5.92	1.58e-08
Seattle - Frau I	2.02	3.82	0.53	0.98
Seattle - Frau II	4.54	3.82	1.19	0.76
Seattle - HANC	28.17	3.82	7.38	1.11e-12
Seattle - Opt HANC	5.57	3.82	1.46	0.59

Table AI: Percent of scatter gated events that are live CD45+

CellType	Condition	Mean	95% CI	n
CD45	Fresh	10.32	[8.61, 12.03]	24
CD45	Frozen	15.59	[12.75, 18.42]	24

Table AJ: Percent of scatter gated events that are live CD45+. Difference in percentage from fresh, paired t-test

CellType	Condition	Mean	95% CI	p	n
CD45	Frozen	5.262	[3.68, 6.84]	5.09e-07	24

Cryopreservation of endocervical cytobrushes

Table AK: Average recovery

CellType	Condition	Mean	95% CI	n
CD3	Cocktail	60.87	[37.81, 83.94]	13
CD3	DMSO	53.31	[37.65, 68.96]	13
CD14	Cocktail	66.22	[41.65, 90.8]	13
CD14	DMSO	67.74	[39.2, 96.28]	13
CD66b	Cocktail	31.20	[19.7, 42.7]	13
CD66b	DMSO	36.15	[11.48, 60.81]	13

Table AL: Effect size relative to 10% DMSO, paired t-test

CellType	Condition	Mean	95% CI	p	n
CD3	Cocktail	7.564	[-6.11, 21.24]	0.25	13
CD14	Cocktail	-1.514	[-10.92, 7.9]	0.73	13
CD66b	Cocktail	-4.947	[-29.24, 19.34]	0.67	13

Table AM: Number of live cells (log10, if whole sample had been treated this way)

CellType	Condition	Mean	95% CI	n
CD14	Cocktail	3.945	[3.61, 4.29]	13
CD14	DMSO	3.977	[3.68, 4.28]	13
CD3	Cocktail	3.657	[3.32, 4]	13
CD3	DMSO	3.635	[3.34, 3.93]	13
CD66b	Cocktail	5.261	[4.95, 5.58]	13
CD66b	DMSO	5.260	[4.95, 5.57]	13

Cryopreservation of whole cytobrushes

Table AN: Number of live cells (log10)

CellType	Condition	Mean	95% CI	n
CD3	Isolated cells	3.084	[2.04, 4.13]	6
CD3	Whole brush	3.273	[2.53, 4.01]	6
CD14	Isolated cells	2.919	[2.09, 3.75]	6
CD14	Whole brush	2.983	[2.15, 3.82]	6
CD66b	Isolated cells	3.928	[3.09, 4.77]	6
CD66b	Whole brush	4.084	[3.2, 4.97]	6

Table AO: Difference in cell numbers (log10) relative to isolated cells, paired t-test

CellType	Condition	Mean	95% CI	p	n
CD3	Whole brush	0.18909	[-0.28, 0.66]	0.35	6
CD14	Whole brush	0.06437	[-0.73, 0.86]	0.84	6
CD66b	Whole brush	0.15621	[-0.79, 1.11]	0.69	6

Table AP: Viability

CellType	Condition	Mean	95% CI	n
CD3	Isolated cells	68.48	[53.9, 83.07]	6
CD3	Whole brush	68.28	[51.95, 84.61]	6
CD14	Isolated cells	75.27	[59.72, 90.82]	6
CD14	Whole brush	60.05	[40.36, 79.74]	6
CD66b	Isolated cells	60.27	[42.63, 77.92]	6
CD66b	Whole brush	39.68	[12.23, 67.14]	6

Table AQ: Absolute viability relative to isolated cells, paired t-test

CellType	Condition	Mean	95% CI	p	n
CD3	Whole brush	-0.2042	[-5.92, 5.51]	0.93	6
CD14	Whole brush	-15.2208	[-32.97, 2.53]	0.08	6
CD66b	Whole brush	-20.5915	[-47.58, 6.4]	0.11	6

Cryopreservation of colorectal immune cells

Table AR: Average recovery

CellType	Condition	Mean	95% CI	n
CD3	6% DMSO 5% EG 6% HES	43.61	[26.08, 61.15]	4
CD3	GHRC I	52.64	[33.2, 72.08]	4
CD3	GHRC II	52.93	[35.07, 70.79]	4
CD3	HANC	56.71	[40, 73.43]	4
CD3	Optimal HANC	54.88	[34.46, 75.3]	4
CD8	6% DMSO 5% EG 6% HES	41.80	[27.09, 56.51]	4
CD8	GHRC I	52.32	[35.3, 69.35]	4
CD8	GHRC II	50.77	[37.4, 64.13]	4
CD8	HANC	57.50	[43.09, 71.91]	4
CD8	Optimal HANC	55.10	[36.96, 73.24]	4
CD13	6% DMSO 5% EG 6% HES	38.73	[3.92, 73.54]	4
CD13	GHRC I	47.11	[2.09, 92.13]	4
CD13	GHRC II	44.98	[2.1, 87.86]	4
CD13	HANC	46.78	[5.5, 88.05]	4
CD13	Optimal HANC	48.15	[-0.55, 96.85]	4
CD33	6% DMSO 5% EG 6% HES	24.19	[16.58, 31.8]	4
CD33	GHRC I	30.90	[21.61, 40.2]	4
CD33	GHRC II	28.28	[20.21, 36.35]	4
CD33	HANC	34.55	[24.68, 44.41]	4
CD33	Optimal HANC	33.59	[19.28, 47.89]	4
CD206	6% DMSO 5% EG 6% HES	30.63	[15.14, 46.12]	4
CD206	GHRC I	41.72	[19.97, 63.48]	4
CD206	GHRC II	35.35	[15.44, 55.27]	4
CD206	HANC	43.83	[17.17, 70.49]	4
CD206	Optimal HANC	44.18	[12.71, 75.65]	4
CD66b	6% DMSO 5% EG 6% HES	14.12	[2.93, 25.32]	4
CD66b	GHRC I	16.75	[8.67, 24.84]	4
CD66b	GHRC II	16.36	[5.51, 27.21]	4
CD66b	HANC	18.28	[7.3, 29.25]	4
CD66b	Optimal HANC	17.39	[6.67, 28.11]	4

Table AS: CD3 recovery, repeated measures ANOVA with Tukey post-test

Comparisons	Estimate	Std. Error	Z value	p
GHRC I - 6% DMSO 5% EG 6% HES	9.03	1.62	5.58	1.60e-07
GHRC II - 6% DMSO 5% EG 6% HES	9.32	1.62	5.77	7.59e-08
GHRC II - GHRC I	0.29	1.62	0.18	1
HANC - 6% DMSO 5% EG 6% HES	13.1	1.62	8.1	2.44e-15
HANC - GHRC I	4.07	1.62	2.52	0.09
HANC - GHRC II	3.78	1.62	2.34	0.13
Optimal HANC - 6% DMSO 5% EG 6% HES	11.27	1.62	6.97	2.86e-11
Optimal HANC - GHRC I	2.24	1.62	1.39	0.64
Optimal HANC - GHRC II	1.95	1.62	1.2	0.75
Optimal HANC - HANC	-1.83	1.62	-1.13	0.79

Table AT: CD13 recovery, repeated measures ANOVA with Tukey post-test

Comparisons	Estimate	Std. Error	Z value	p
GHRC I - 6% DMSO 5% EG 6% HES	8.39	2.7	3.1	0.02
GHRC II - 6% DMSO 5% EG 6% HES	6.26	2.7	2.31	0.14
GHRC II - GHRC I	-2.13	2.7	-0.79	0.93
HANC - 6% DMSO 5% EG 6% HES	8.05	2.7	2.98	0.02
HANC - GHRC I	-0.34	2.7	-0.12	1
HANC - GHRC II	1.79	2.7	0.66	0.96
Optimal HANC - 6% DMSO 5% EG 6% HES	9.42	2.7	3.49	4.65e-03
Optimal HANC - GHRC I	1.04	2.7	0.38	1
Optimal HANC - GHRC II	3.17	2.7	1.17	0.77
Optimal HANC - HANC	1.37	2.7	0.51	0.99

Table AU: Change in cytokine production relative to fresh, paired t-test

Stim	Cytokine	Mean	95% CI	p	n
CEF	Any	1.1467	[-1.6, 3.9]	0.214747	3
CEF	CD107a	0.8233	[-2.4, 4.05]	0.386602	3
CEF	IFNg	0.7967	[-2.48, 4.08]	0.405500	3
CEF	IL2	0.5867	[-2.07, 3.24]	0.441986	3
CEF	MIP	1.0533	[-3.35, 5.46]	0.411585	3
CEF	TNFa	1.0300	[-2.5, 4.56]	0.335880	3
PMA/Iono	Any	8.0440	[-4.94, 21.03]	0.160655	5
PMA/Iono	CD107a	-0.5420	[-17.5, 16.41]	0.933551	5
PMA/Iono	IFNg	18.0100	[2.02, 34]	0.035260	5
PMA/Iono	IL2	24.5680	[10.82, 38.31]	0.007694	5
PMA/Iono	MIP	13.7760	[-7.91, 35.46]	0.152555	5
PMA/Iono	TNFa	19.9940	[6.02, 33.97]	0.016502	5
SEB	Any	2.6720	[-5.95, 11.3]	0.438250	5
SEB	CD107a	1.0300	[-7.29, 9.35]	0.748493	5
SEB	IFNg	2.4580	[-3.94, 8.86]	0.346324	5
SEB	IL2	2.8660	[-1.12, 6.85]	0.116320	5
SEB	MIP	3.0280	[-6.81, 12.86]	0.440833	5
SEB	TNFa	4.9860	[1.15, 8.82]	0.022525	5
