# Supplementary material for: Cryopreservation of Human Mucosal Leukocytes
Source: PLoS One. 2016 May 27;11(5):e0156293. doi: 10.1371/journal.pone.0156293 (PMC4883784; doi:10.1371/journal.pone.0156293)
Supplement: S2 File — Contains the complete R code and raw data needed to reproduce the analyses reported here, as well as generate all figures. (ZIP) [file pone.0156293.s003.zip › Supporting File S4 - Analysis code and data/Supporting File S4 - Analysis code and data/data/Data README.docx]

Overview of the data

This document describes the data files. Note that the code includes functions for each data file that reformat the data, sometimes dramatically. The data are included as is for fuller transparency, but using the getData() functions may be more useful.

data/cryopreservation-mixtures.csv: Effect of mixtures of CPAs on viability and recovery after cryopreservation in vaginal cells.

| Variable | Values | Description |
| --- | --- | --- |
| Experiment | 14-20 | Experiment number |
| DMSOConcentration | 0  10%  2.50%  4%  5%  6%  7%  7.50%  8%  9% | Concentration of dimethylsulfoxide (% v/v) |
| EGConcentration | 0  10%  2.50%  5%  7.50% | Concentration of ethylene glycol (% v/v) |
| TrehaloseConcentration | 0  200mM  50mM | Concentration of trehalose, (mM) |
| CellType | CD14  CD3 | Type of cell |
| Viability | 50.1-96 | Viability (%, max 100) |
| CellCount | 782-13337 | Number of cells |

data/cryopreservation-with-trehalose.csv: Effect of CPA-trehalose mixtures on viability and recovery after cryopreservation in vaginal cells.

| Variable | Values | Description |
| --- | --- | --- |
| Experiment | 7-20 | Experiment number |
| CPA | Control  DMSO  EG  None  PG | Cryoprotective agent |
| Concentration | 0  0.75M  1.25M  1.5M  1.67%  1.75M  10%  11.67%  12%  13.33%  14%  1M  2M  3.33%  5%  6%  7%  7.50%  8%  8.33%  9% | Concentration of CPA (% v/v or M, as indicated) |
| TrehaloseConcentration | 0-1575 | Concentration of trehalose, (mM) |
| CellType | CD14  CD3 | Type of cell |
| Viability | 5.45-96 | Viability (%, max 100) |
| CellCount | 406-13337 | Number of cells |

data/cryopreservation.csv: Effect of single CPAs on viability after cryopreservation in vaginal cells.

| Variable | Values | Description |
| --- | --- | --- |
| Experiment | 1-6 | Experiment number |
| CPA | Control  DMSO  EG  Glycerol  PG | Cryoprotective agent |
| Concentration | 0  0.75M  1.25M  1.5M  1.75M  10%  1M  2M  5%  6%  7%  8%  9% | Concentration of CPA (% v/v or M, as indicated) |
| CellType | CD14  CD3 | Type of cell |
| Viability | 5.02-95.7 | Viability (%, max 100) |

data/cytobrush-whole.csv: Comparison of cryopreserving cytobrushes on the brush or after isolating the cells on viability and cell number in vaginal cells.

| Variable | Values | Description |
| --- | --- | --- |
| Sample | 4999-5123 | Sample identifier |
| Method | Isolated cells  Whole brush | Cells frozen on the cytobrush or in suspension |
| Brush.number | 1-2 | Whether this brush was the first or second taken from the participant |
| CellType | All live cells  Live Macrophages  Live Neutrophils  Live T cells | Type of cell |
| Percent.live | 6.94-88.09 | Viability (%, max 100) |
| AbsCounts | 69.97-819739.73 | Number of cells |

data/cytobrushes.csv: Effect of different cryopreservation media on viability and recovery after cryopreservation of cytobrushes.

| Variable | Values | Description |
| --- | --- | --- |
| Sample | 001-2  008-2  009-2  011-2  1  11  12  14  27  28  29  3  30  31  32  33  35  4  6  9 | Sample identifier |
| Method | Cocktail  DMSO  ST | Cryopreservation medium (  cocktail = 6% DMSO, 5% EG, 50 mM trehalose  DMSO = 10% DMSO  ST = SmartTube, not analyzed here) |
| Neutro | 143-1040000 | Number of neutrophils |
| Macro | 2.07-28756 | Number of macrophages |
| T | 12.4-17423 | Number of T cells |
| LiveNeutro | 62-547000 | Number of live neutrophils |
| LiveMacro | 0-13730 | Number of live macrophages |
| LiveT | 12.4-14687 | Number of live T cells |
| NeutroRecov | 0.64-165.38 | Recovery of neutrophils (%) |
| MacroRecov | 0-222.09 | Recovery of macrophages (%) |
| TRecov | 0.95-228.23 | Recovery of T cells (%) |
| NeutroViab | 0.62-92.7 | Viability of neutrophils (%, max 100) |
| MacroViab | 0-91.77 | Viability of macrophages (%, max 100) |
| TViab | 6.06-100 | Viability of T cells (%, max 100) |

data/formal-comparison.csv: Formal validation of new cryopreservation medium and comparison to two other cryopreservation media.

| Variable | Values | Description |
| --- | --- | --- |
| TissueID | 242-261 | Sample identifier |
| Condition | 10D  DT  Mix  Unfrozen | Whether and how sample was cryopreserved (  10D = 10% DMSO  DT = 8% DMSO and 50 mM trehalose  Mix = 6% DMSO, 5% EG, 50 mM trehalose) |
| CellType | CD14  CD3 | Type of cell |
| CellCount | 714-30346 | Number of cells |
| Viability | 42.7-93.4 | Viability (%, max 100) |

data/objective-1-cd45.csv: Effect of cryopreservation on percentage of cells that are CD45+ in vaginal cells.

| Variable | Values | Description |
| --- | --- | --- |
| TissueID | 278-329 | Sample identifier |
| Condition | Filoceth  Fraunhofer I  Fraunhofer II  HANC  HANC A  HANC as Fraunhofer  HANC B  HANC C  HANC D  HANC slow add  HANC thawed as Fraunhofer  Roederer  Seattle fast  Seattle Fast  Seattle Fraunhofer  Seattle Fraunhofer 0.5C  Seattle Fraunhofer 2.5C  Seattle Fraunhofer 5C  Seattle Fraunhofer BSA  Seattle Fraunhofer HES  Seattle Fraunhofer HES BSA  Seattle Fraunhofer HES BSA 0.5C  Seattle Fraunhofer HES BSA 2.5C  Seattle Fraunhofer HES BSA 3.5C  Seattle Fraunhofer HES BSA 3C  Seattle Fraunhofer HES BSA 5C  Seattle Fraunhofer HES BSA set 2  Seattle Fraunhofer HES BSA set 2 10C  Seattle Fraunhofer old EG  Seattle slow  Seattler Fraunhofer  Unfrozen FBS  Unfrozen FI  Unfrozen FII  Unfrozen Filoceth  Unfrozen Fraunhofer I  Unfrozen Fraunhofer II | Whether and how sample was cryopreserved (see table at end of this document for explanation of values) |
| PercentCD45 | 0.15-40.2 | Percentage of scatter gated, live cells that are CD45+ |

data/objective-1.csv: Comparison of published and novel cryopreservation media, as well as different processing methods in vaginal cells.

| Variable | Values | Description |
| --- | --- | --- |
| TissueID | 278-329 | Sample identifier |
| Condition | Filoceth  Fraunhofer I  Fraunhofer II  HANC  HANC A  HANC as Fraunhofer  HANC B  HANC C  HANC D  HANC slow add  HANC thawed as Fraunhofer  Roederer  Seattle fast  Seattle Fraunhofer  Seattle Fraunhofer 0.5C  Seattle Fraunhofer 2.5C  Seattle Fraunhofer 5C  Seattle Fraunhofer BSA  Seattle Fraunhofer HES  Seattle Fraunhofer HES BSA  Seattle Fraunhofer HES BSA 0.5C  Seattle Fraunhofer HES BSA 2.5C  Seattle Fraunhofer HES BSA 3.5C  Seattle Fraunhofer HES BSA 3C  Seattle Fraunhofer HES BSA 5C  Seattle Fraunhofer HES BSA set 2  Seattle Fraunhofer HES BSA set 2 10C  Seattle Fraunhofer old EG  Seattle slow  Unfrozen FBS  Unfrozen Filoceth  Unfrozen Fraunhofer I  Unfrozen Fraunhofer II | Whether and how sample was cryopreserved (see table at end of this document for explanation of values) |
| CPA | 10% DMSO  6% DMSO 5% EG 50 mM trehalose  6% DMSO 5% EG 50 mM trehalose in RPMI/BSA  6% DMSO 5% EG 6% HES  6% DMSO 5% EG 6% HES in FBS  6% DMSO 5% EG 6% HES in RPMI/BSA  Filoceth  Fraunhofer I  Fraunhofer II  Unfrozen | Cryoprotective agent |
| EG | new  old | Lot of ethylene glycol |
| CellType | CD14  CD3 | Type of cell |
| AbsoluteViability | 41.35-96.7 | Absolute viability (%, max 100) |
| Recovery | 16.58-130.79 | Recovery (%) |
| Viability | 58.38-109.06 | Relative viability (%, relative to fresh cells) |
| FreezingRate | 0.5-10 | Rate of cooling during freezing (degrees C per min) |
| subtractedHANCRecovery | -23.14-83.42 | Recovery with HANC recovery subtracted |
| subtractedHANCViability | -23.84-25.88 | Viability with HANC viability subtracted |

data/Sinclair_CD45_counts.csv: Comparison of different methods of cryopreserving colorectal cells, absolute cell numbers.

| Variable | Values | Description |
| --- | --- | --- |
| TissueID | 1247-3610 | Sample identifier |
| ViableCD45Per4.4Biopsies | 718000-2480000 | Number of viable CD45 cells per sample |
| Condition | Fraunhofer I  Fraunhofer II  Fresh  HANC  HANC as Fraunhofer  Seattle | Whether and how sample was cryopreserved (see table at end of this document for explanation of values) |

data/Sinclair_Percents.csv: Comparison of different methods of cryopreserving colorectal cells, percents of CD45.

| Variable | Values | Description |
| --- | --- | --- |
| TissueID | 1247-3610 | Sample identifier |
| CD3 | 0.26-0.56 | Fraction of viable CD45+ cells that were CD3+ (max 1) |
| CD8 | 0.06-0.41 | Fraction of viable CD45+ cells that were CD8+ (max 1) |
| CD13 | 0.05-0.22 | Fraction of viable CD45+ cells that were CD13+ (max 1) |
| CD33 | 0.03-0.11 | Fraction of viable CD45+ cells that were CD33+ (max 1) |
| CD206 | 0.01-0.06 | Fraction of viable CD45+ cells that were CD206+ (max 1) |
| CD66b | 0-0.03 | Fraction of viable CD45+ cells that were CD66b+ (max 1) |
| Condition | Fraunhofer I  Fraunhofer II  Fresh  HANC  HANC as Fraunhofer  Seattle | Whether and how sample was cryopreserved (see table at end of this document for explanation of values) |

data/UCSF-polyfunctionality.csv: Cytokine production by colorectal cells fresh or after cryopreservation.

| Variable | Values | Description |
| --- | --- | --- |
| Subject | 1517-7234 | Sample identifier |
| HIV Status | Negative  Positive | HIV status |
| Cell Type | CD8+ | Type of cell |
| Stimulation | CEF  PMA/Iono  SEB | Type of stimulation |
| Tissue Type | Fresh GALT  Frozen GALT Suspension | Type of sample |
| 7+F+2+M+T+ | 0-51.93 | Percentage of cells positive for this population. Column name describes cytokines included in this population  7 = CD107a  F = IFNγ  2 = IL-2  M = MIP-1β  T = TNFα |
| 7+F+2+M+T- | 0-12.38 |  |
| 7+F+2+M-T+ | 0-4.26 |  |
| 7+F+2+M-T- | 0-0.25 |  |
| 7+F+2-M+T+ | 0-11.17 |  |
| 7+F+2-M+T- | 0-23.75 |  |
| 7+F+2-M-T+ | 0-1.56 |  |
| 7+F+2-M-T- | 0-1.44 |  |
| 7+F-2+M+T+ | 0-1.82 |  |
| 7+F-2+M+T- | 0-0.48 |  |
| 7+F-2+M-T+ | 0-1.32 |  |
| 7+F-2+M-T- | 0-0.17 |  |
| 7+F-2-M+T+ | 0-1.84 |  |
| 7+F-2-M+T- | 0-7.56 |  |
| 7+F-2-M-T+ | 0-2.51 |  |
| 7+F-2-M-T- | 0-19.83 |  |
| 7-F+2+M+T+ | 0-37.5 |  |
| 7-F+2+M+T- | 0-10.25 |  |
| 7-F+2+M-T+ | 0-5.52 |  |
| 7-F+2+M-T- | 0-1.43 |  |
| 7-F+2-M+T+ | 0-5.09 |  |
| 7-F+2-M+T- | 0-9.61 |  |
| 7-F+2-M-T+ | 0-1.1 |  |
| 7-F+2-M-T- | 0-1.85 |  |
| 7-F-2+M+T+ | 0-4.01 |  |
| 7-F-2+M+T- | 0-1.47 |  |
| 7-F-2+M-T+ | 0-3.33 |  |
| 7-F-2+M-T- | 0-0.94 |  |
| 7-F-2-M+T+ | 0-2.33 |  |
| 7-F-2-M+T- | 0-11.41 |  |
| 7-F-2-M-T+ | 0-6.37 |  |
| 7-F-2-M-T- | 4.84-100 |  |

*Explanation of cryopreservation conditions. This table defines the conditions used in Sinclair_Percents.csv, Sinclair_CD45_counts.csv, objective-1.csv, and objective-1-cd45.csv.*

| Name | Cryopreservation medium | Procedure | Freezing rate |
| --- | --- | --- | --- |
| Filoceth | Filoceth (see PubMed ID 22580762 ) | Add CPA like HANC, thaw like Fraunhofer | 1 |
| Fraunhofer I | 10% DMSO in RPMI/12.5% BSA | B | 1 |
| Fraunhofer II | 5% DMSO, 6% HES in RPMI/12.5% BSA | B | 1 |
| HANC | 10% DMSO in FBS | HANC | 1 |
| HANC A | 10% DMSO in FBS | HANC but wash with 10 mL in 15 mL tube | 1 |
| HANC as Fraunhofer | 10% DMSO in FBS | B | 1 |
| HANC B | 10% DMSO in FBS | HANC but 3 min CPA addition in ice water bath | 1 |
| HANC C | 10% DMSO in FBS | HANC but only 1 post-thaw wash | 1 |
| HANC D | 10% DMSO in FBS | HANC but no benzonase | 1 |
| HANC slow add | 10% DMSO in FBS | HANC but 3 min CPA addition in ice water bath | 1 |
| HANC thawed as Fraunhofer | 10% DMSO in FBS | HANC but 1 post-thaw wash in 10 mL in 15 mL tube | 1 |
| Roederer | 10% DMSO in FBS | HANC | 1 |
| Seattle fast | 6% DMSO 5% EG 1.2% trehalose in FBS | HANC | 1 |
| Seattle Fraunhofer | 6% DMSO 5% EG 1.2% trehalose in FBS | B | 1 |
| Seattle Fraunhofer 0.5C | 6% DMSO 5% EG 1.2% trehalose in FBS | B | 0.5 |
| Seattle Fraunhofer 2.5C | 6% DMSO 5% EG 1.2% trehalose in FBS | B | 2.5 |
| Seattle Fraunhofer 5C | 6% DMSO 5% EG 1.2% trehalose in FBS | B | 5 |
| Seattle Fraunhofer BSA | 6% DMSO 5% EG 1.2% trehalose in RPMI/12.5% BSA | B | 1 |
| Seattle Fraunhofer HES | 6% DMSO 5% EG 6% HES in FBS | B | 1 |
| Seattle Fraunhofer HES BSA | 6% DMSO 5% EG 6% HES in RPMI/12.5% BSA | B | 1 |
| Seattle Fraunhofer HES BSA 0.5C | 6% DMSO 5% EG 6% HES in RPMI/12.5% BSA | B | 0.5 |
| Seattle Fraunhofer HES BSA 2.5C | 6% DMSO 5% EG 6% HES in RPMI/12.5% BSA | B | 2.5 |
| Seattle Fraunhofer HES BSA 3.5C | 6% DMSO 5% EG 6% HES in RPMI/12.5% BSA | B | 3.5 |
| Seattle Fraunhofer HES BSA 3C | 6% DMSO 5% EG 6% HES in RPMI/12.5% BSA | B | 3 |
| Seattle Fraunhofer HES BSA 5C | 6% DMSO 5% EG 6% HES in RPMI/12.5% BSA | B | 5 |
| Seattle Fraunhofer HES BSA set 2 | 6% DMSO 5% EG 6% HES in RPMI/12.5% BSA | B | 1 |
| Seattle Fraunhofer HES BSA set 2 10C | 6% DMSO 5% EG 6% HES in RPMI/12.5% BSA | B | 10 |
| Seattle Fraunhofer old EG | 6% DMSO 5% EG 1.2% trehalose in FBS | B | 1 |
| Seattle slow | 6% DMSO 5% EG 1.2% trehalose in FBS | A | 1 |
| Unfrozen FBS | NA | Unfrozen | NA |
| Unfrozen Filoceth | NA | Unfrozen | NA |
| Unfrozen Fraunhofer I | NA | Unfrozen | NA |
| Unfrozen Fraunhofer II | NA | Unfrozen | NA |
