# Supplementary material for: Cryopreservation of Human Mucosal Leukocytes
Source: PLoS One. 2016 May 27;11(5):e0156293. doi: 10.1371/journal.pone.0156293 (PMC4883784; doi:10.1371/journal.pone.0156293)
Supplement: S2 File — Contains the complete R code and raw data needed to reproduce the analyses reported here, as well as generate all figures. (ZIP) [file pone.0156293.s003.zip › Supporting File S4 - Analysis code and data/Supporting File S4 - Analysis code and data/README.html]

README


# README

#### *Sean Hughes*

This directory holds the code and data used to generate the figures and statistics presented in the accompanying manuscript. This directory is assumed to be the working directory. A brief summary of the files follows:

- `figures.R` generates the figures
- `statistics.Rmd` generates the statistical tables
- `clean-cpas.R` normalizes the units used for the different cryopreservation media
- `getData.R` loads data for particular experiments and does some data cleaning and processing as needed
- `library-all.R` loads all required packages
- `plotStyles.R` has helper functions for consistent styling of figures
- `source-all.R` sources all files necessary for the figures and statistics to be generated
- `statisticalUtils.R` has helper functions for consistent statistical tables
- `utils.R` has miscellaneous helper functions

All of the data reside in the `/data/` directory and are most easily accessed through the functions in `getData.R`.

To recreate all of the figures, simply `source("figures.R")`. This will create all the figures as individual image files in `/figures/`. To recreate the statistical tables, knit the `statistics.Rmd` file with `knitr`. This will create `statistics.pdf` in the top level directory.

Figures, statistics, and this file were generated on 2016-03-23, using the following packages and versions:

```
## Session info --------------------------------------------------------------
```

```
##  setting  value                       
##  version  R version 3.2.3 (2015-12-10)
##  system   x86_64, mingw32             
##  ui       RTerm                       
##  language (EN)                        
##  collate  English_United States.1252  
##  tz       America/Los_Angeles         
##  date     2016-03-23
```

```
## Packages ------------------------------------------------------------------
```

```
##  package      * version date       source        
##  acepack        1.3-3.3 2013-05-03 CRAN (R 3.2.0)
##  assertthat     0.1     2013-12-06 CRAN (R 3.2.0)
##  cluster        2.0.3   2015-07-21 CRAN (R 3.2.3)
##  codetools      0.2-14  2015-07-15 CRAN (R 3.2.3)
##  colorspace     1.2-6   2015-03-11 CRAN (R 3.2.0)
##  DBI            0.3.1   2014-09-24 CRAN (R 3.2.0)
##  devtools       1.10.0  2016-01-23 CRAN (R 3.2.3)
##  digest         0.6.9   2016-01-08 CRAN (R 3.2.3)
##  dplyr        * 0.4.3   2015-09-01 CRAN (R 3.2.3)
##  evaluate       0.8.3   2016-03-05 CRAN (R 3.2.4)
##  foreign        0.8-66  2015-08-19 CRAN (R 3.2.3)
##  formatR        1.3     2016-03-05 CRAN (R 3.2.4)
##  Formula      * 1.2-1   2015-04-07 CRAN (R 3.2.0)
##  ggplot2      * 2.0.0   2015-12-18 CRAN (R 3.2.3)
##  gridExtra      2.2.1   2016-02-29 CRAN (R 3.2.3)
##  gtable       * 0.1.2   2012-12-05 CRAN (R 3.2.0)
##  Hmisc        * 3.16-0  2015-04-30 CRAN (R 3.2.0)
##  htmltools      0.3     2015-12-29 CRAN (R 3.2.3)
##  knitr          1.12.3  2016-01-22 CRAN (R 3.2.3)
##  lattice      * 0.20-33 2015-07-14 CRAN (R 3.2.3)
##  latticeExtra   0.6-28  2016-02-09 CRAN (R 3.2.3)
##  lazyeval     * 0.1.10  2015-01-02 CRAN (R 3.2.0)
##  magrittr       1.5     2014-11-22 CRAN (R 3.2.0)
##  memoise        1.0.0   2016-01-29 CRAN (R 3.2.3)
##  multcomp     * 1.4-3   2016-02-03 CRAN (R 3.2.3)
##  munsell        0.4.2   2013-07-11 CRAN (R 3.2.0)
##  mvtnorm      * 1.0-3   2015-07-22 CRAN (R 3.2.1)
##  nlme         * 3.1-124 2016-01-20 CRAN (R 3.2.3)
##  nnet           7.3-11  2015-08-30 CRAN (R 3.2.3)
##  pander       * 0.6.0   2015-11-23 CRAN (R 3.2.3)
##  plyr           1.8.3   2015-06-12 CRAN (R 3.2.1)
##  proto          0.3-10  2012-12-22 CRAN (R 3.2.0)
##  R6             2.1.2   2016-01-26 CRAN (R 3.2.3)
##  RColorBrewer   1.1-2   2014-12-07 CRAN (R 3.2.0)
##  Rcpp           0.12.3  2016-01-10 CRAN (R 3.2.3)
##  readr        * 0.2.2   2015-10-22 CRAN (R 3.2.3)
##  reshape2     * 1.4.1   2014-12-06 CRAN (R 3.2.3)
##  rmarkdown      0.9.2   2016-01-01 CRAN (R 3.2.3)
##  rpart          4.1-10  2015-06-29 CRAN (R 3.2.3)
##  sandwich       2.3-4   2015-09-24 CRAN (R 3.2.3)
##  scales         0.3.0   2015-08-25 CRAN (R 3.2.3)
##  stringi        1.0-1   2015-10-22 CRAN (R 3.2.3)
##  stringr      * 1.0.0   2015-04-30 CRAN (R 3.2.3)
##  survival     * 2.38-3  2015-07-02 CRAN (R 3.2.1)
##  TH.data      * 1.0-6   2015-01-05 CRAN (R 3.2.1)
##  tidyr        * 0.4.1   2016-02-05 CRAN (R 3.2.3)
##  yaml           2.1.13  2014-06-12 CRAN (R 3.2.0)
##  zoo            1.7-12  2015-03-16 CRAN (R 3.2.1)
```
