# Supplementary material for: Cryopreservation of Human Mucosal Leukocytes
Source: PLoS One. 2016 May 27;11(5):e0156293. doi: 10.1371/journal.pone.0156293 (PMC4883784; doi:10.1371/journal.pone.0156293)
Supplement: S1 Text — (DOCX) [file pone.0156293.s005.docx]

Cryopreservation of mucosal immune cells

Contents

[Cryopreservation procedure 2](#_Toc443395434)

[Cryopreservation media and resuspension solutions 3](#_Toc443395435)

[Reagent sources 4](#_Toc443395436)

[References 5](#_Toc443395437)

## Cryopreservation procedure

Reagents

1. Prepare 2X cryopreservation medium and resuspension solution and refrigerate for >1h (see “Cryopreservation media and resuspension solutions” section below).
   1. Should be prepared the day of cryopreservation.
2. Label cryovials and refrigerate for >1h.

Cryopreservation

1. Suspend cells at up to 40 million/mL in resuspension solution.
2. Place a cryovial rack into an ice water bath and add the chilled cryovials. Adjust the ice water level to submerge the bottom third of the cryovials.
3. Aliquot up to 0.5 mL cell suspension to each cryovial.
4. Over 2-3 minutes, add an equal volume of the 2X cryopreservation medium to the cryovial drop-by-drop (e.g. to 0.5 mL cell suspension in resuspension solution, add 0.5 mL of the 2X CPA)^[[1]](#footnote-1)^.
5. Place cryovials in a Mr. Frosty or other 1^o^C per minute freezing device and transfer to -80^o^C freezer.
6. After >2h, transfer on dry ice or liquid nitrogen to the vapor phase of a liquid nitrogen freezer.

Thawing

1. For each cryovial to be thawed, add 10 mL of cell culture medium to a 15 mL tube. Place the tube in a 37^o^C water bath for >30 minutes.
   1. If desired, include benzonase at a concentration of 50 U/mL.^[[2]](#footnote-2)^
   2. Note: using a 15 mL tube instead of a 50 mL tube gives greater recovery.
2. Remove cryovials from the freezer and hold on liquid nitrogen until ready to thaw.
3. Transfer 15 mL tubes from the water bath to a biosafety cabinet.
4. Transfer up to four cryovials to a 37^o^C water bath. Swirl until ice smaller than a pea remains in each tube.
5. Dropwise, add 1 mL of the media from the 15 mL tubes to each of the cryovials over ~30 seconds (add one drop to the first cryovial, then one drop to the next cryovial, and so on.)
6. Transfer the contents of each cryovial to a 15 mL tube containing media. Rinse out the empty cryovial with 1 mL from the same 15 mL tube and return media to the 15 mL tube.
7. Centrifuge the tubes at 300*g* for 10 minutes, resuspend with fresh media or other buffer, and use as desired.
   1. Note: washing once instead of twice gives greater recovery.

## Cryopreservation media and resuspension solutions

**Introduction:** We found the following four cryopreservation media to yield equivalent recoveries of mucosal immune cells [1,2]. Medium A is the standard medium used for PBMC cryopreservation. Media B and D have reduced DMSO concentrations and media C and D don’t require the use of serum. Medium B was developed for this study. Media C and D were previously developed [3]. Note that Medium B appears worse for colorectal leukocytes than the other media.

**Medium A: 10% DMSO in FBS**

*Resuspension solution (FBS):*

1 mL fetal bovine serum

*2X cryopreservation medium (20% v/v DMSO in FBS):*

200 µL DMSO, 800 µL FBS

**Medium B: 6% DMSO, 5% EG, 6% HES in FBS**

*Resuspension solution (FBS):*

1 mL FBS

*2X cryopreservation medium (12% v/v DMSO, 10% v/v EG, 12% w/v HES in FBS):*

120 µL DMSO, 100 µL EG, 780 µL FBS/HES stock solution

*Notes:*

Prepare FBS/HES stock solution (15.4% w/v HES) by dissolving 0.77 g HES in 4.5 mL FBS in a water bath. After dissolution, bring final volume to 5 mL with FBS and filter sterilize. Store at 4^o^C for up to one month.

**Medium C: GHRC I (10% DMSO in RPMI with 12.5% BSA)**

*Resuspension solution (12.5% w/v BSA in RPMI):*

500 µL BSA/RPMI stock solution, 500 µL RPMI

*2X cryopreservation medium (20% v/v DMSO in 12.5% w/v BSA in RPMI):*

200 µL DMSO, 500 µL BSA/RPMI stock solution, 300 µL RPMI

*Notes:*

Prepare BSA/RPMI stock solution (25% w/v BSA) by dissolving 1.25 g BSA in 4 mL RPMI in a water bath. After dissolution, bring final volume to 5 mL with RPMI and filter sterilize. Store at 4^o^C for up to one month.

**Medium D: GHRC II (5% DMSO, 6% HES in RPMI with 12.5% BSA)**

*Resuspension solution (6% w/v HES, 12.5% w/v BSA in RPMI):*

250 µL HES/RPMI solution, 500 µL BSA/RPMI stock solution, 250 µL RPMI

*2X cryopreservation medium (10% v/v DMSO, 6% w/v HES in 12.5% w/v BSA in RPMI):*

100 µL DMSO, 250 µL HES/RPMI stock solution, 500 µL BSA/RPMI stock solution, 150 µL RPMI

*Notes:*

Prepare HES/RPMI solution (24% w/v HES) by dissolving 1.2 g HES in 4.5 mL RPMI in a water bath. After dissolution, bring final volume to 5 mL with RPMI and filter sterilize. Store at 4^o^C for up to one month.

Prepare BSA/RPMI stock solution as described for GHRC I.

## Reagent sources

- Dimethyl sulfoxide (e.g. Sigma-Aldrich D2650)
- Ethylene glycol (e.g. Sigma-Aldrich 324558)
- Bovine serum albumin (e.g. Sigma-Aldrich A9647)
- Hydroxyethyl starch (AK Scientific V0118 http://www.aksci.com/item_detail.php?cat=V0118)
- RPMI-1640 with HEPES (your favorite brand)
- Fetal bovine serum (your favorite brand)

## References

1. Shu Z, Hughes SM, Fang C, Huang J, Fu B, Zhao G, et al. A Study of the Osmotic Characteristics, Water Permeability, and Cryoprotectant Permeability of Human Vaginal Immune Cells. Cryobiology. 2016;In press.

2. Shu Z, Hughes SM, Fang C, Hou Z, Zhao G, Fialkow M, et al. Determination of the membrane permeability to water of human vaginal mucosal immune cells at subzero temperatures using differential scanning calorimetry. Biopreserv Biobank. 2016;In press.

3. Germann A, Schulz JC, Kemp-Kamke B, Zimmermann H, von Briesen H. Standardized Serum-Free Cryomedia Maintain Peripheral Blood Mononuclear Cell Viability, Recovery, and Antigen-Specific T-Cell Response Compared to Fetal Calf Serum-Based Medium. Biopreserv Biobank. 2011;9: 229–236. doi:10.1089/bio.2010.0033

1. Using an appropriate rack and multichannel pipettor (the kind where the spacing of the tips can be adjusted, e.g., Pipet-Lite XLS Adjustable-spacer or by skipping tips on a static multichannel), this can be done for 6-8 cryovials at a time. [↑](#footnote-ref-1)
2. Benzonase digests nucleic acids and can prevent cell clumping caused by DNA released from dead cells. [↑](#footnote-ref-2)
